# Supplementary material for: AANAT1 regulates insect midgut detoxification through the ROS/CncC pathway
Source: Commun Biol. 2024 Jul 3;7:808. doi: 10.1038/s42003-024-06505-x (PMC11222512; doi:10.1038/s42003-024-06505-x)
Supplement: Supplementary file 2 — Supplementary Information [file 42003_2024_6505_MOESM2_ESM.pdf]

Supplementary Table 1. Primers used in this study.

**The primers for Real-time PCR**	**Upper primer (5'-3')**	**Lower primer (5'-3')**
BdorAANAT 1	GAAGGATAAAGAGGCGGTGC	AGATGGCGAGGGTCTTTTCA
LOC105225815 (GstD1)	TCGGCATTGAGTTGAACAAG	GTCGGTCTTGGCATATTGCT
LOC105225817 (GstD1)	ACTATTTGCCTGCTTCCGCT	GGGGTCTTTCGGGTACAAGG
LOC109579500 (Gst1)	CAAGAAGCGTGCTGTCATCA	AGTTGATCACCCGCCACATA
LOC115066421 (GstE14)	GGAAGCATTTAAAGCCGTTCG	TTCCACGAGTGCTTCCAAAC
LOC109579784 (GstE4)	CCGCCAGTCAACAAGTTCTT	AGTAAAACAGCACGACACGG
LOC105229681 (GstE9)	CGGGTGTATTGTTCCAAGGC	CGGCTAGAGTGATGGTCTGT
LOC105229682 (GstE7)	CGGCTTTATTGGATGATGGT	CGCTCAAAGGCCATACTAGC
LOC105222104 (GstD1)	CGCCCTGTCGCTCTATTCTA	CCTCGGCCAAGTAGATGAGT
LOC105225363 (Cyp9b2)	CTTCGTAAACCACACACCGT	CATCAAACCAAACATGGCGC
LOC105227474 (Cyp6a2)	AAACAAACGGCGCATCAAAC	GGCGTACGTTCGAACACAAA
LOC105228201 (Cyp6a13)	TGTGGGCGCCTTATATTGGA	CTTGAGGAAAGTGTAGGCGC
LOC105230935 (Cyp6d5)	AAACGTGAAGCAGCATTGGG	CCACCACTGAATTGCGGATC
LOC105233810 (Cyp6g2)	ATGGATGCGAAAACCAACTC	CCCGAAAAGCTTAACCATCA
LOC105224660 (UGT36-D1)	CGCCATTCATAGGGTTGAGC	GATAGCGGGCGTGTTGAAAT
LOC115065792 (UGT49-C1)	TACGGCCACTTTTGCTCAAC	GCTGGGGAATTTGATTGGCA
LOC105230268 (UGT35)	GGGTTGTACAGATGGGACGA	AGTGATTTCATGACCGCGTG
LOC105230828 (UGT301-D1)	CACGTAACGGCACTCACAAA	ATGAGCGCTTTCACTTTGGG
Bdor-CncC	CGCTACTGAGTCACAATGGC	TGTGCATATGTGACAGCTGC
Bdpr-Maf	CCCGACATAAGCGACGATGA	GACAGCTAGCCGCATAACCA
Bdor-Keap1	TTGCGATGTGCTACGAAAAG	TCGGGGAATTCGTAAGTTTG
Bdor-α-Tubulin	CGCATTCATGGTTGATAACG	GGGCACCAAGTTAGTCTGGA
Bd-RpL32	CCCGTCATATGCTGCCAACT	GCGCGCTCAACAATTTCCTT
AaAANAT1	GTAGCCATTTCTCTGCCCGA	AACCGCAGTGTGTGGTTTCT
AaCncC	CGCTACCGTCAGCTACCAAT	CGGCTCCCTCTAAGTGACTG
AaMaf	GAAGAACCGGGGATATGCGG	CTTCCTCAGCGCTTCGTACT
AaKeap1	CGACGGAAAAAGAGCATAGC	GGCGCCATATTGTTTCTTGT
AaS7	GGGACAAATCGGCCAGGCTATC	TCGTGGACGCTTCTGCTTGTTG
**The primers for *AaAANAT1* Cloning**	**Upper primer (5'-3')**	**Lower primer (5'-3')**
AaAANAT1-ORF Cloning	ATGGAAATCGAAATTCAAAATCAC	TTAGGCCAGTCTTTTTGTTAGA
**The primers for dual-luciferase reporter gene assay**	**Upper primer (5'-3')**	**Lower primer (5'-3')**
Bd-CncC-ORF-complete	ATGGGTTTGCCCGCTTCGGA	TCAATCTTTCTGATGCGTTT
BdCncC-homologous recombination	tacgactcactataggaaggATGGGTTTGCCCGCTTCGGAG	ccatcgtggtctttatagtcATCTTTCTGATGCGTTTGATGATGATGTGCG
LOC105225363-promter	ctatcgataggtaccgagctcGTTTTTGGACGCCCAGGAC	cagtaccggaatgccaagcttATAAACAAGGTGTATTTGTTTTGCACT
LOC105225815-promter	ctatcgataggtaccgagctcTTTCTTCAAATTTCTTGCGAAATC	cagtaccggaatgccaagcttAATTTACTTAAATAGACACACAGTCATGAGC
LOC105227474-promter	ctatcgataggtaccgagctcAATGTAGTTGA	cagtaccggaatgccaagcttGGACGTTG

	AAAGAATTTAGAGCGA	CTATTCTCCTTCTTTAT
LOC105228201-promter	ctatcgataggtaccgagctcGAAATTCTTTCC GGATCACATCA	cagtaccggaatgccaagcttAACTGACT TTGCAAATGCATTTTG
LOC105229682-promter	ctatcgataggtaccgagctcTTTGTATATTAG CTATTTATACTCTGCGC	cagtaccggaatgccaagcttGTACTTATG TATGCATGTATCTGAAATGG
LOC105230935-promter	ctatcgataggtaccgagctcTAACATTTTGTT GCTTCCTTTTATAGC	cagtaccggaatgccaagcttAAATGGTC CTCATCACTGAACAATAT
LOC105233810-promter	ctatcgataggtaccgagctcCGCTTGCTTTAG CGCTTTTAT	cagtaccggaatgccaagcttTATTTGAAA AAATATTTCGTTTTATAGATTTT
LOC115065792-promter	ctatcgataggtaccgagctcACCTATCGCTAG CAGCGCAC	cagtaccggaatgccaagcttTTTGGCCAT TTTTTTCTCTTATATGG
LOC115066421-promter	ctatcgataggtaccgagctcTTTGCGCTATTT GTAGAGAAATTTATAC	cagtaccggaatgccaagcttATCGTGCCG CGTGAAAAG
LOC105222104-promter	ctatcgataggtaccgagctcGAAAATGAAAA CTCTCACACATCGA	cagtaccggaatgccaagcttTGTTATAAT TTCGTCAATTTAATGGACG
**The primers for double-strand RNA synthesis**	**Upper primer (5'-3')**	**Lower primer (5'-3')**
GFP-RNAi	***TAATACGACTCACTATAGGG***AAGGG CGAGGAGCTGTTCACCG	***TAATACGACTCACTATAGGG***CA GCAGGACCATGTGATCGCGC
BdorAANAT1-RNAi	***TAATACGACTCACTATAGGG***TAAAT TTCTGGATTTGGGTG	***TAATACGACTCACTATAGGG***CG GTGTGAACACCACTTCAT
BdorCncC-RNAi	***TAATACGACTCACTATAGGG***TCGAT GGAGCAACGTTTACA	***TAATACGACTCACTATAGGG***TGT CCATCAAATGCAAATCC
AaAANAT1-RNAi	***TAATACGACTCACTATAGGG***TAATT ACGGAAGACGACGCC	***TAATACGACTCACTATAGGG***TTC CATTACTCGGGCAGAGA

Supplementary Table 2. **Susceptibility of adults of WT and *AANAT*$^{10/10}$ strains of *B.dorsalis* to diverse insecticides.**

Insecticide	Strain	LC_{50} (mg/L)	95% FL (mg/L)	Toxicty ratio
Chlorpyrifos	WT	11.749	11.246-12.303	2.04
	AANAT1$^{10/10}$	5.754	5.346-6.194	
Trichlorfon	WT	1.373	1.263-1.483	2.91
	AANAT1$^{10/10}$	0.472	0.445-0.500	
Deltamethrin	WT	8.09	6.09-8.99	1.88
	AANAT1$^{10/10}$	4.31	3.17-5.73	
Cyclaniliprole	WT	24.54	22.37-27.10	1.72
	AANAT1$^{10/10}$	14.27	10.94-18.03	
Triflumezopyrim	WT	20.63	16.35-26.19	1.74
	AANAT1$^{10/10}$	11.89	9.90-13.93	
Fluralaner	WT	3.60	2.77-5.04	2.20
	AANAT1$^{10/10}$	1.64	1.32-2.03	
Spinosad	WT	34.80	27.60-43.34	3.94
	AANAT1$^{10/10}$	8.84	6.07-12.51	
Emamectin-benzoate	WT	38.77	33.88-44.20	2.05
	AANAT1$^{10/10}$	18.90	15.90-22.36	
Tetraniliprole	WT	29.68	25.67-34.64	3.01
	AANAT1$^{10/10}$	9.86	8.75-11.07	
Spirotetramat	WT	44.05	38.05-51.99	1.99
	AANAT1$^{10/10}$	22.14	17.62-28.40	
Thiamethoxam	WT	4.49	3.674-5.481	1.45
	AANAT1$^{10/10}$	3.09	2.404-3.882	

Toxicity ratio was calculated as dividing LC_{50} of WT by that of *AANAT1*$^{10/10}$. LC50 values were considered significantly different if their fiducial limits did not overlap.

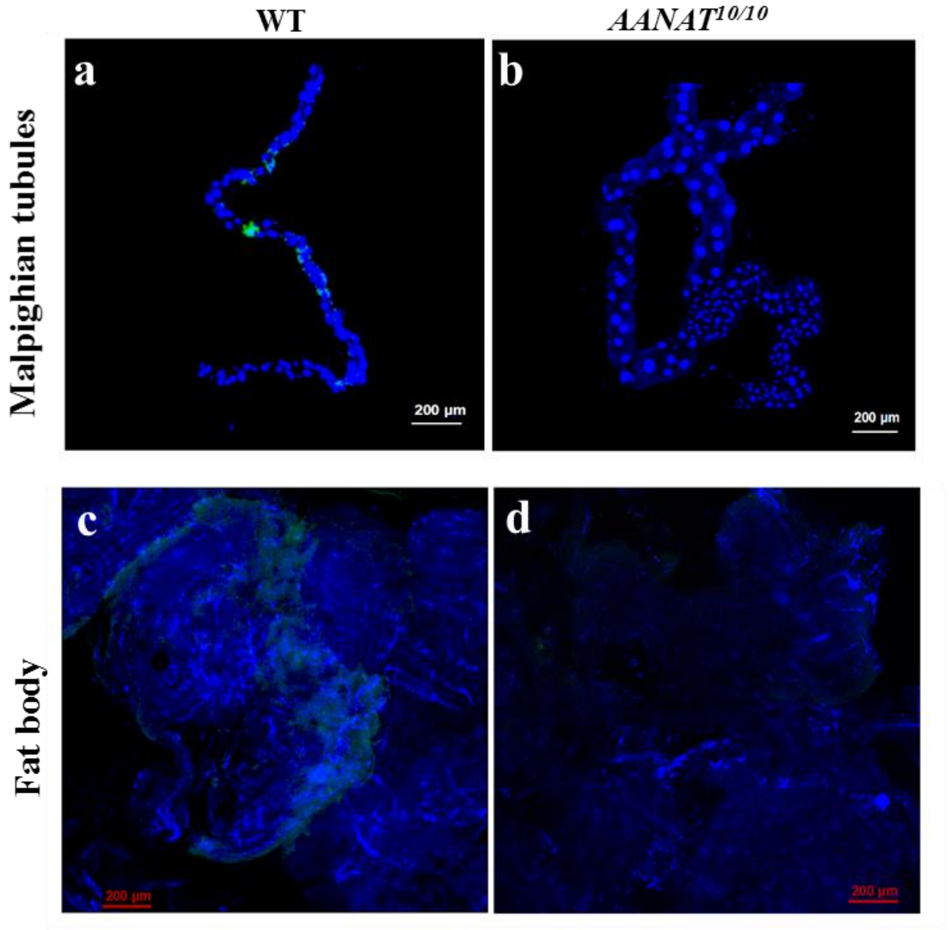

Supplementary Figure 1. Immunostaining of *B. dorsalis* Malpighian tubules (a and b) and fatbody (c and d) using BdorAANAT1 antibody.

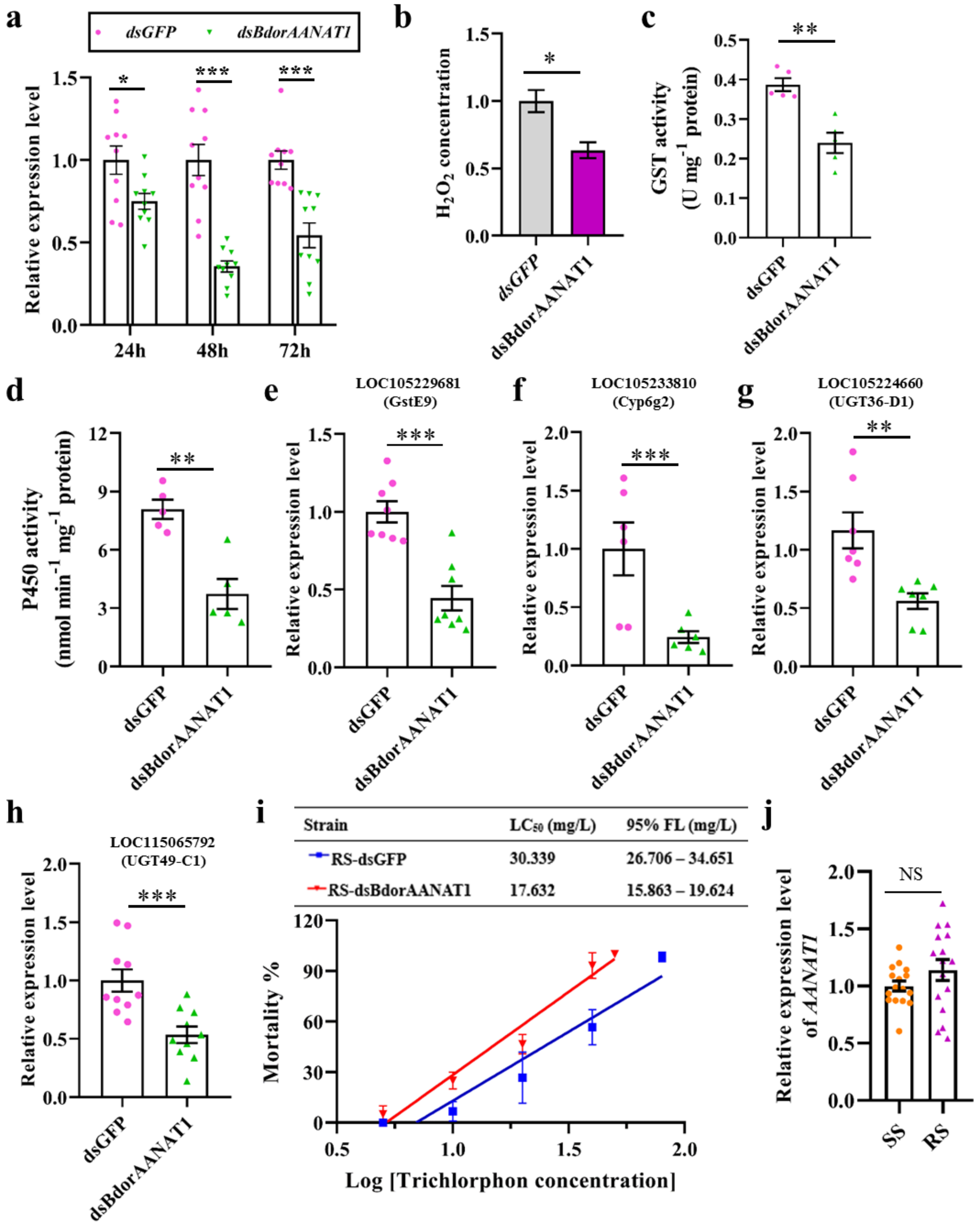

Supplementary Figure 2. Effect of *BdorAANAT1* knockdown on midgut detoxification. (**a**) Midgut *BdorAANAT1* silencing efficiency post injected with dsGFP or ds*AANAT1*. (**b**) Effects of *BdorAANAT1* knockdown on midgut H_2O_2 concentration. Data of H_2O_2 level were normalized to controls. (**c** and **d**) Regulation of both GST and P450 activities in the midguts of *AANAT1*-silencing flies. (**e-h**) Transcriptional responses of *LOC105229681 (GstE9)*,

LOC105233810 (Cyp6g2), *LOC105224660 (UGT36-D1)*, and *LOC115065792 (UGT49-C1)* to *BdorAANAT1* knockdown. (**i**) Effect of *BdorAANAT1* knockdown on the resistant strain (RS) flies to trichlorphon. (**j**) Transcripts level of *BdorAANAT1* in the midgut of sensitive strain (SS) and resisitant strain (RS) flies. LC_{50}, lethal concentration that kills 50% of *B. dorsalis* adults; 95% FL, 95% fiducial limits of LC_{50}. Student's t test was performed for **a-h**, and **j**. Error bars indicate ± s.e.m., ****p*<0.001, ***p*<0.01, **p*<0.05. All results were repeated in at least two independent experiments. LC_{50} values were considered significantly different if their fiducial limits did not overlap.

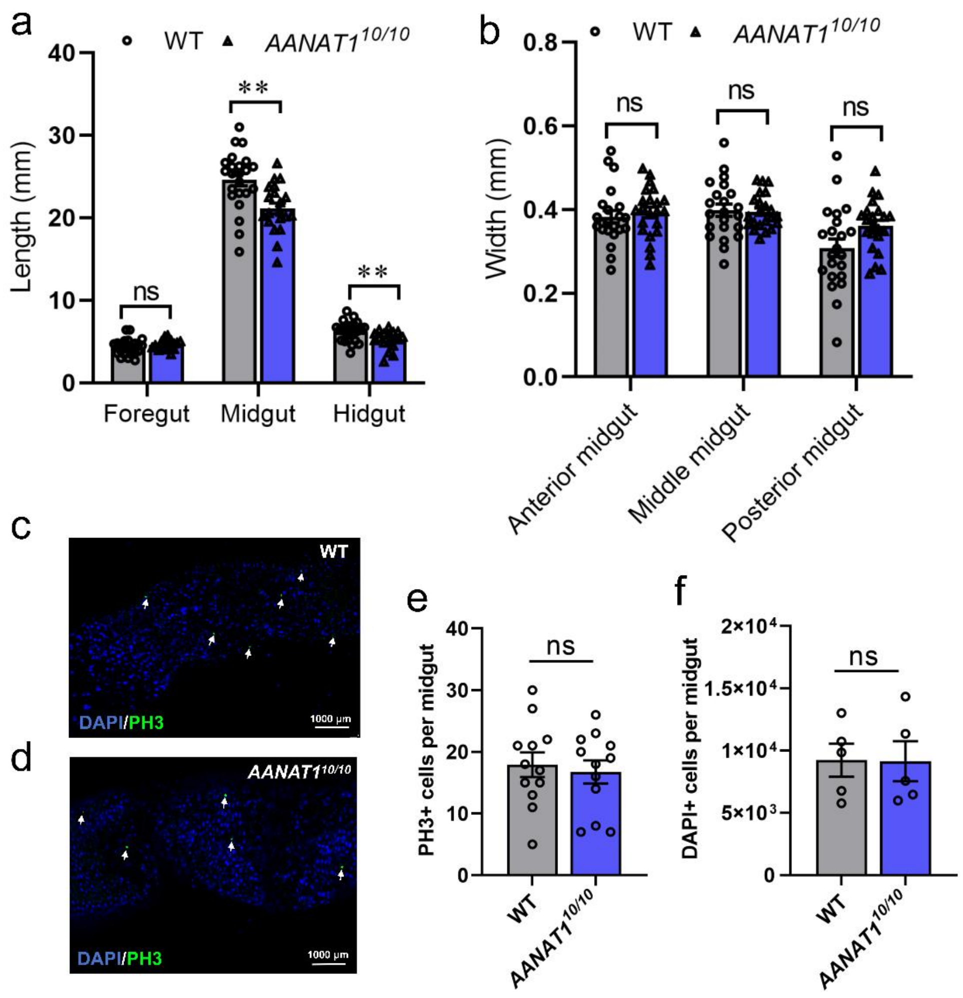

Supplementary Figure 3. Gut development of WT and *AANAT1*$^{10/10}$ mutants. (**a**) Comparison of the developmental length of the foregut, midgut, and hindgut between the WT and *AANAT1*$^{10/10}$ mutant flies. (**b**) Comparison of the width of the anterior, middle, and posterior

midgut of WT and *AANAT1*$^{10/10}$ mutant flies. (**c** and **d**) The 15-day-old wild-type (**c**) and *AANAT1*$^{10/10}$ (**d**) flies were stained with anti-PH3 (green) and DAPI (blue). All PH3 positive cells are indicated by arrows. (**e** and **f**) The number of PH3-positive cells (**e**) and DAPI-positive cells (**f**) in the midgut of WT and *AANAT1*$^{10\text{-}10}$ mutant flies were counted. Data were analyzed by Student's t test. Error bars indicate ±s.e.m.; ****p*<0.001, ***p*<0.01, **p*<0.05.

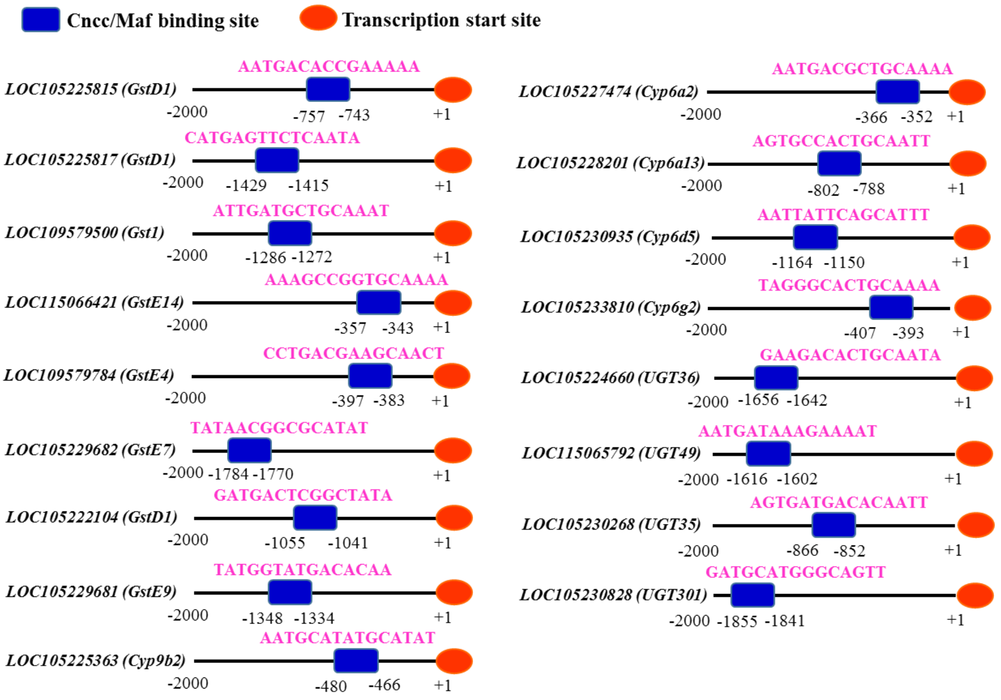

Supplementary Figure 4. Prediction of cap 'n' collar isoform C – muscle aponeurosis fibromatosis (CncC–Maf) binding site in the promoter regions of identified detoxification genes in *B. dorsalis*.

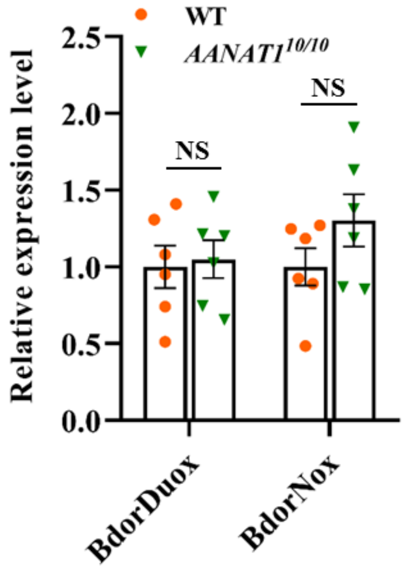

Supplementary Figure 5. Expression level of *Duox* and *Nox* in the midguts of WT and *AANAT1*$^{10/10}$ strain flies. All values were normalized to *Rpl32* expression. Data represent mean ± SEM for biological replicates, with a two-tailed Student's t test, ns, not significant ($p > 0.05$).

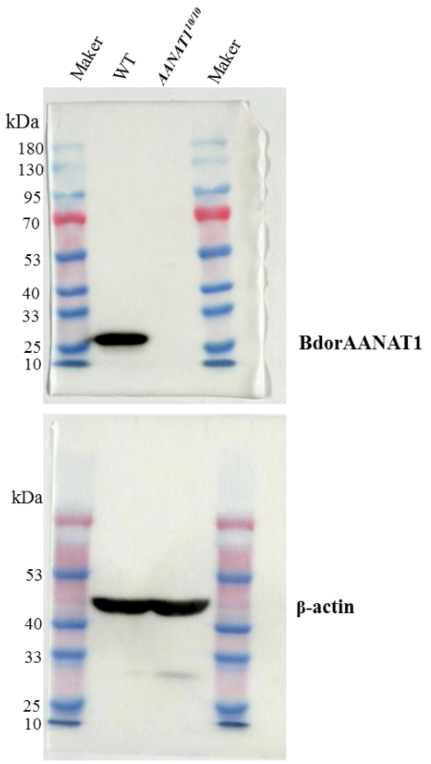

Supplementary Figure 6. Uncropped blot images for Figure 1b.

Supplementary Methods

Gut development

To compare the intestinal development between WT and *AANAT1*$^{10/10}$ mutant strains, we adapted a method based on *Drosophila* gut length measurement[1]. Intact intestines, comprising the foregut, midgut, and hindgut, of 10-day-old flies were dissected in phosphate-buffered saline (PBS). Subsequently, they were fixed in 4% paraformaldehyde for 1 hour, followed by imaging under a stereomicroscope (SMZ18, Nikon). Images were captured, and intestinal length and midgut width were measured using built-in software functionalities (n=22). Lengths of the foregut, midgut, and hindgut were individually assessed. The width was measured via dividing the midgut into anterior, middle, and posterior regions. Each region was measured three times. Average value was calculated to determine the width of the respective midgut segment.

To investigate the effect of *AANAT1*$^{10\text{-}10}$ knockout on midgut cell proliferation, we used DAPI and PH3 cell staining[2]. Immunostaining was performed as described in main manuscript. Briefly, midguts were fixed in 4% % paraformaldehyde for 2 h at room temperature, washed in PBST, blocked for 1 h at room temperatureat, and roomincubated with rabbit antiphospho-histone H3 (Beyotime Biotech) in blocking solution overnight at 4℃. The samples were then washed in PBST, incubated with goat anti-rabbit IgG Alexa Fluor Plus 488 for 2 h at room temperature, washed, and incubated DAPI for 5 min. Finally, samples were mounted with kisse's mounting medium. The images were observed with a Leica Stellaris 5 confocal microscope (Leica, Wetzlar, Germany), and analyzed them with ImageJ software.

Supplementary References

1. Christensen CF, et al. (2024) *Drosophila* activins adapt gut size to food intake and promote regenerative growth. *Nature Communications* 15(1): 273.

2. Lee SH, et al. (2012) Requirement of matrix metalloproteinase-1 for intestinal homeostasis in the adult *Drosophila* midgut. *Experimental Cell Research* 318(5): 670-681.
